# Construction of a prognostic glycolysis‐related lncRNA signature for patients with colorectal cancer

**DOI:** 10.1002/cam4.4851

**Published:** 2022-05-26

**Authors:** Xinyang Zhong, Xuefeng He, Yaxian Wang, Zijuan Hu, Huixia Huang, Senlin Zhao, Hong Zhang, Ping Wei, Dawei Li

**Affiliations:** ^1^ Department of Colorectal Surgery Fudan University Shanghai Cancer Center Shanghai China; ^2^ Department of Oncology Shanghai Medical College Fudan University Shanghai China; ^3^ Department of Pathology Fudan University Shanghai Cancer Center Shanghai China; ^4^ Cancer Institute, Fudan University Shanghai Cancer Center Shanghai China; ^5^ Institute of Pathology Fudan University Shanghai China; ^6^ Colorectal Tumor Surgery Ward, Department of General Surgery Shengjing Hospital of China Medical University Shenyang People's Republic of China

**Keywords:** colorectal cancer, glycolysis, lncRNA, prognosis

## Abstract

Aerobic glycolysis is a common metabolic phenotype in tumors that helps cancer cells adjust to severe living conditions and can aid metastasis in several types of carcinomas, including colorectal cancer (CRC). Long non‐coding RNAs (lncRNAs) can influence tumor biology and have been previously used to assess patients' outcomes and to identify potential therapeutic targets. However, despite the importance of glycolysis‐related lncRNAs (GRLs) in the development of CRC, studies on their use as prognostic markers are still limited. Herein, we applied a series of bioinformatic analyses to screen potential prognostic lncRNAs for colorectal cancer. Out of all lncRNAs screened, nine GRLs were selected to constitute a prognostic signature. Based on the signature, two molecular subtypes were classified with distinct prognostic outcomes and excellent diagnostic accuracy (The 1‐, 3‐ and 5‐year AUC are 0.756, 0.716, and 0.721, respectively). The prognostic value of this signature was further validated using another cohort. The enriched molecular pathways, immune infiltration, and mutation landscape were also significantly different between the two groups. The different drug sensitivity results between the two groups suggest a potential strategy for precise treatment. Furthermore, we confirmed that AFAP1‐AS1 could regulate aerobic glycolysis and metastasis of CRC cells. Overall, we developed a glycolysis‐related lncRNA (GRL) signature and suggested that this signature could offer a predictive value and identify potential therapeutic targets for cancer therapy.

## Graphical abstract

In this article, a glycolysis‐related lncRNA signature was constructed in colorectal cancer and this signature is correlated with patients’ survival and clinicopathological features. Furthermore, the relevant signalling pathways and immune infiltration landscape as identified by our model may pave the way for new therapeutic strategies for CRC. Finally, we demonstrated that AFAP1‐AS1 could regulate aerobic glycolysis and be a promising target in CRC.

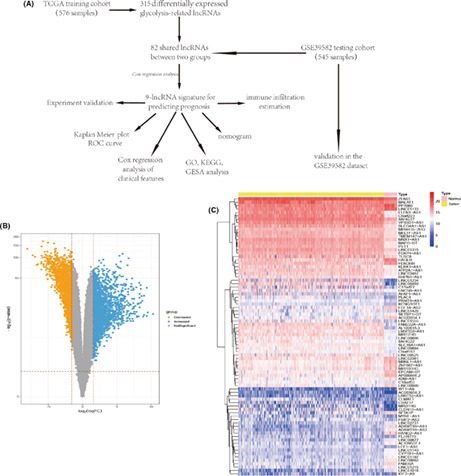

## 1 INTRODUCTION

Colorectal cancer (CRC) is the third most common cancer and constitutes approximately 10% of the annual total diagnosed malignancies and cancer‐related deaths globally.^1^, ^2^ Although novel strategies, such as targeted therapy, have been recently developed at a rapid pace, an estimate of only 14% of metastatic CRC patients could survive the disease for 5 years.^2^, ^3^ Alarmingly, both the incidence and death rates of young patients less than 65 years of age have increased in the past few years.^2^ Multiple risk factors including lifestyle, obesity, genetic, and environmental factors have been associated with CRC.^3^, ^4^ However, the exact reasons for the increase in incidence and death rates are not completely understood.

Energy metabolism reprogramming is one of the hallmarks of cancer.^5^ In the early 20th century, Nobel laureate Otto H. Warburg observed that tumor cells depend on the high throughput of glycolysis to meet their energy requirements regardless of cellular oxygen levels, also known as “Warburg effect.”^6^, ^7^ The Warburg effect or aerobic glycolysis helps tumors adjust to severe living conditions, leading to cancer progression. Our previous research indicated that a high level of aerobic glycolysis mediated by the FOXC1/FBP1 signaling axis can accelerate CRC progression.^8^ The indispensable role of aerobic glycolysis in colorectal cancer makes it a potential therapeutic target to prevent metastasis.^9^ Tumors exhibit high levels of glycolysis to gain a competitive advantage against other cells in environments with limited metabolic resources.^10^, ^11^, ^12^ Besides producing lactic acid, glycolysis is also regarded as an adaptative mechanism for synthesizing biomacromolecules to meet urgent growth requirements. Glucose can be used as a carbon source for anabolic processes such as in the de novo synthesis of nucleotides, lipids, and proteins, leading to an immunosuppressive tumor microenvironment (TME).^13^, ^14^, ^15^

Long noncoding RNAs (lncRNAs) can promote CRC progression through numerous mechanisms, including but not limited to influencing metastatic TME and remodeling the metabolic features of cancer cells, thus making them potential therapeutic targets for cancer treatment.^16^, ^17^ Recently we reported that the lncRNA MIR17HG could accelerate CRC metastasis through HK1‐mediated upregulation of glycolysis activity.^18^ The expression of lncRNAs varies in different types and stages of cancers, demonstrating their predictive power in evaluating the survival and disease progression of patients with CRC.^19^ Glycolysis has close interaction with lncRNA expression, suggesting that glycolysis‐related lncRNAs (GRLs) can be used to construct a signature with predictive value.^20^ For instance, a signature consisting of three GRLs was found to be correlated with metastasis and patients' survival in clear cell renal cell carcinoma.^21^ Similarly, Tong et al. set up a glycolysis‐related lncRNA (GRL) signature which divided ovarian cancer patients into two subgroups with distinct prognosis and immune microenvironment.^22^ Immune checkpoint inhibitors (ICIs) have been approved to treat CRC and have provided reliable responses and disease control in patients with mismatch repair deficiency (MMR‐D) and/or microsatellite instability‐high (MSI‐H).^3^, ^23^ However, most patients do not benefit from immunotherapy—a phenomenon which might be attributed to an immunosuppressive TME. In relation to this, it has been shown that lncRNAs are important in TME remodeling and can affect the infiltration landscape of CRC, suggesting that lncRNA expression can reflect the TME of CRC and guide immunotherapy.^24^, ^25^ The relationship between TME and GRL signature was explored in some types of cancers.^21^, ^22^ However, relevant research is absent in CRC. Hence, we constructed a GRL model which could forecast patients' survival and provide promising therapeutic guidance for managing CRC. In addition, we screened a novel GRL, AFAP1‐AS1, and experimentally validated its oncogenic function in CRC.

## 2 METHODS AND MATERIALS

### 2.1 Datasets preparation

The RNA sequencing and related clinical data of 576 CRC tumor samples and 51 adjacent normal samples were obtained from The Cancer Genome Atlas (TCGA) database (https://cancergenome.nih.gov). The overall survival (OS) of the patients included in this research was identified to be >30 days. The GSE39582 dataset was obtained from the Gene Expression Omnibus (GEO) database (https://www.ncbi.nlm.nih.gov/geo/query), which includes 545 CRC samples (OS >30 days) with relevant clinical data.

### 2.2 Selection of the differentially expressed glycolysis‐related lncRNAs

Differentially expressed lncRNAs between paracancerous and cancerous tissues were identified by the R package “edgeR.” Hits with a |log2 fold change| > 1.5 and *P* value < 0.05 were considered as differentially expressed lncRNAs. Glycolysis‐related genes were obtained from the Molecular Signatures Database (MSigDB) (http://www.gsea‐msigdb.org/gsea/msigdb). Differentially expressed glycolysis‐related lncRNAs (GRLs) were identified using Pearson analysis (|R| > 0.3 and *P* < 0.001).

### 2.3 Construction of a GRL signature

Differentially expressed GRLs that were correlated with the OS of CRC patients in TCGA cohort were screened using univariate Cox regression analysis (*P* < 0.05). Then, a least absolute shrinkage and selection operator (LASSO) Cox regression analysis was carried out in R using the “glmnet” package. Through multivariate Cox regression analysis, the regression coefficient of each candidate lncRNA was obtained. Risk scores were calculated using this mathematical formula: [coefficient(a) × expression(a)] + [coefficient(b) × expression(b)] + …. + [coefficient(n) × expression(n)].

### 2.4 LncRNA‐mRNA expression network

All glycolysis‐related genes that exhibited a correlation with the lncRNAs in our signature (|R| > 0.3 and *P* < 0.001) were screened out. Using package “ggalluvial,” a Sankey diagram was drawn to visualize the interaction between lncRNA and mRNA.

### 2.5 Nomogram

A nomogram was used to predict the 1, 3, and 5‐year survival probability of CRC patients. Moreover, the discrimination and accuracy of the nomograms were assessed by calibration curves and concordance index (C‐index).

### 2.6 GO, KEGG, and GSEA analysis

The differentially expressed genes between the high and low‐score groups in the TCGA cohort were subjected to gene ontology (GO) and Kyoto Encyclopedia of Genes and Genomes (KEGG) analysis. As for gene set enrichment analysis (GSEA) analysis,

we downloaded gene set “h.all.v7.4.entrez.gmt” from MsigDB and then compared the.

enriched hallmarks between the two subgroups.

### 2.7 Immune infiltration evaluation

CIBERSORT algorithm can evaluate the relative proportion of 22 immune cells by simply inputting the gene expression matrix of cancer cells.^26^ The immune cell infiltration data used in this research was obtained from TIMER2.0 (http://timer.cistrome.org/).^27^

### 2.8 Mutational landscape analysis

The DNA mutation data used in this research were obtained from the UCSC Xena (https://xena.ucsc.edu/) website.The R package “maftools” was applied to analyze these data.

### 2.9 Drug sensitivity analysis

The IC50 is a parameter that could quantify a certain drug's inhibitory effect in a specific biochemical or biological function. IC50 of drugs including 5‐fluorouracil, elesclomol, DMOG, dasatinib, camptothecin, and AKT inhibitor VIII was predicted using package “pRRophetic.”^28^

### 2.10 Cell culture and siRNA transfection

The human CRC cell line DLD1 was purchased from the Cell Bank of the Chinese Academy of Sciences. The cells were grown and maintained in RPMI 1640 medium (Gibco, USA) that contains 1% penicillin/streptomycin (Gibco, USA), and 10% fetal bovine serum (Invitrogen, USA). All cells were mycoplasma‐free and cultured in a humidified atmosphere with 5% CO2 at 37°C. Small interfering RNAs (siRNAs) against the lncRNA AFAP1‐AS1 and its negative control (Genomeditech, China) were transfected into DLD1 cells using RNAi MAX (Invitrogen) according to the manufacturer's instructions. Table 3 lists the AFAP1‐AS1 siRNA sequences used in this study.

### 2.11 RNA extraction and RT‐qPCR

The TRIzol reagent (Beyotime Biotechnology, China) was used to isolate total RNA. Reverse transcription was performed using ABScript II RT Mix for qPCR (ABclonal, China). Quantitative real‐time PCR (qRT‐PCR) was performed using TB Green® Premix Ex Taq™ II (TaKaRa, Japan). For normalization, the housekeeping gene β‐Actin was selected as an internal control. Table 3 lists all the qPCR primers used in this study.

### 2.12 Protein extraction and western blot analysis

Western blot was performed as previously described.^29^ Proteins were probed using primary antibodies against HK2 (A0994, 1:1000; ABclonal), LDHA (A1146, 1:1000; ABclonal), PKM2 (A19102, 1:1000; ABclonal), β‐actin (ab133626, 1:5000; Abcam).

### 2.13 Cell migration assay

CRC cell line DLD1 was grown in 6‐well plates and was transfected with the corresponding siRNAs. Upon reaching 90% confluency, a 10 μl pipette tip was used to form a scratch wound on the cell layer. Each well was washed using PBS twice. The widths of the wounds were assessed and measured at 0 h and 48 h by microscopy. Experiments were repeated three times.

### 2.14 Transwell assay

A transwell assay was used to measure the invasive ability of DLD1 cells. For this, the upper chamber of the transwell was precoated with Matrigel. Then equal numbers of DLD1 cells suspended in FBS‐free RPMI 1640 medium were seeded into the transwell inserts, while RPMI 1640 medium with 10% FBS was added into the lower chamber. After 48 h, cells were harvested and fixed with 4% paraformaldehyde for half an hour. Then, crystal violet was used to dye the cells purple and cell numbers were recorded under a light microscope. Experiments were repeated three times.

### 2.15 Aerobic glycolysis analysis

The Glucose Uptake‐Glo™ Assay (Promega, Madison, WI) and Lactate‐Glo™ Assay (Promega, Madison, WI) were performed to measure the glucose uptake and lactate production ability of CRC cells.

### 2.16 Statistical analysis

All statistical analyses were conducted using the R software (version 4.0.3). *P* values of <0.05 were considered statistically significant.

## 3 RESULTS

### 3.1 LncRNA selection

**FIGURE 1:**
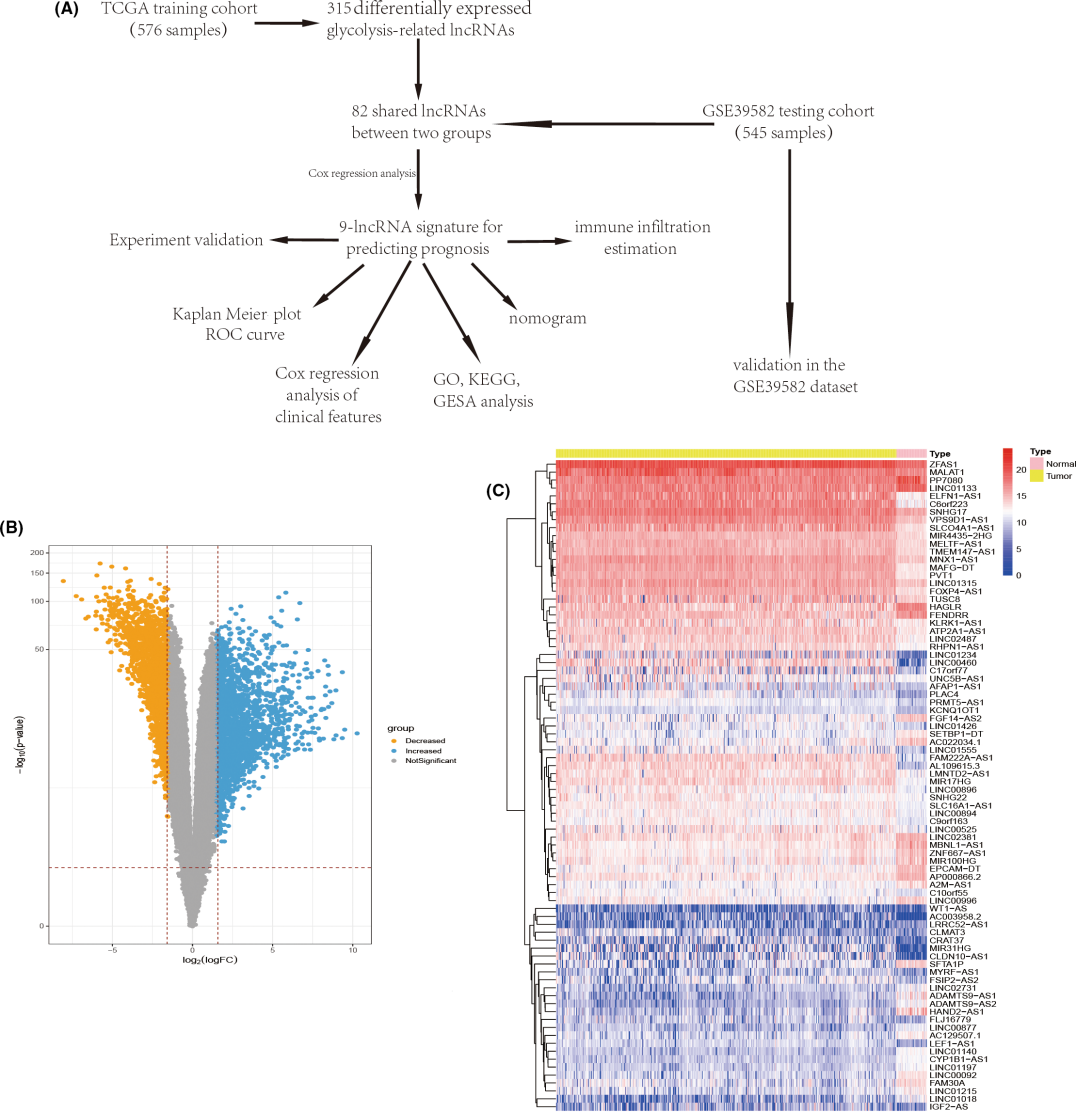
(A) Schematic flowchart of the analysis strategy. (B) The volcano plot shows the distribution of all differentially expressed genes. (C) The heatmap shows the expression of 82 differentially expressed GRLs in both tumor and normal tissues.

This study focused on constructing a glycolysis‐related lncRNA (GRL) signature with prognostic value. A flow diagram which summarizes the overall process used in this study can be seen in Figure 1A. Based on the RNA expression of 576 tumors and 51 adjacent normal samples from CRC patients, 3820 differentially expressed genes were selected using the following criteria: *P* < 0.05 and |log FC| > 1.5 (Figure 1B). From this, we finally selected 533 differentially expressed lncRNAs for downstream analysis. Subsequently, we established a co‐expression network to identify various differentially expressed GRLs based on 219 glycolysis‐related genes from the MSigDB database. Pearson analysis was used and we obtained 315 GRLs (|R| > 0.3 and *P* < 0.001); of which, 82 GRLs were also called out in the GSE39582 dataset. The 82 GRLs identified were represented in a heatmap showing their expression levels in both tumor and adjacent normal tissues (Figure 1C).

### 3.2 Identification of GRL signature in CRC

**FIGURE 2:**
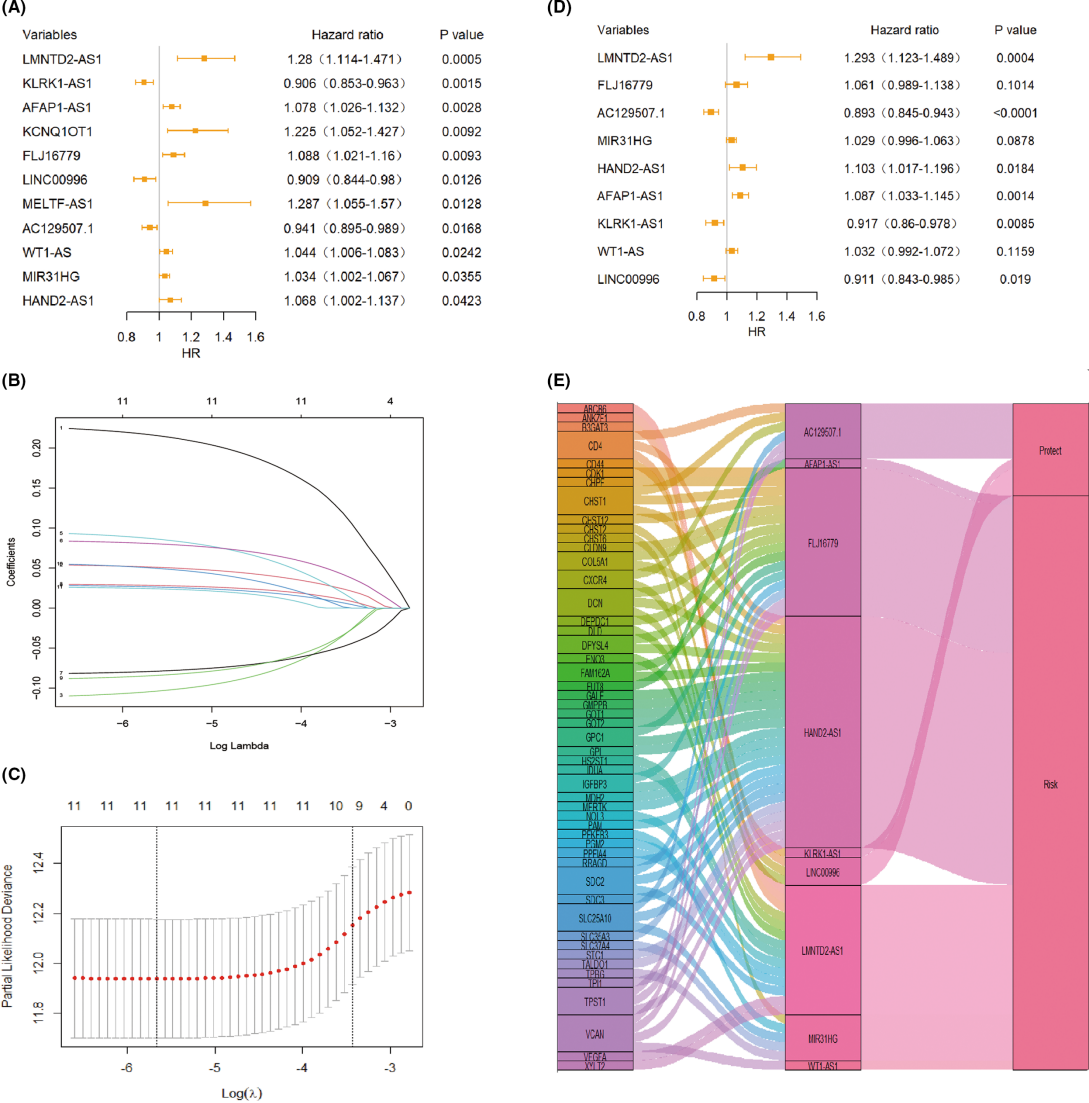
Search for GRLs in colorectal cancer. (A) Univariate Cox regression analysis was applied to confirm that 11 lncRNAs were strongly correlated with patients' overall survival. (B–C) GRL selection utilizing LASSO model. (D) Multivariate Cox regression analysis was applied to confirm the prognostic signature of nine differentially expressed GRLs. (E) Visualization of the relationship between prognostic GRLs, glycolysis‐related genes, and risk types using Sankey's diagram.

Using univariate Cox regression analysis, a list of 11 lncRNAs that were correlated with the OS of CRC patients was finally constructed based on the TCGA dataset (*P* < 0.05; Figure 2A). LASSO Cox regression analysis was applied to further remove 2 lncRNAs based on the value of lambda.1se (Figure 2B,C). Additionally, we performed multivariate Cox regression analysis to calculate the hazard ratio and coefficient of each lncRNA and constructed the prognostic model (Figure 2D). The score was computed based on the following: Score = (LMNTD2‐AS1 * 0.25968 + FLJ16779 × 0.0588 − AC129507.1 × 0.1134 + MIR31HG * 0.02849 + HAND2AS1 × 0.09788 + AFAP1‐AS1 × 0.08383 − KLRK1‐AS1 × 0.0864 + WT1‐AS × 0.03108 − LINC00996 × 0.09303). We used Sankey diagram to further demonstrate the relationship between the prognostic GRLs, glycolysis‐related genes, and risk types (Figure 2E).

### 3.3 GRL signature correlated with prognosis in CRC

**FIGURE 3:**
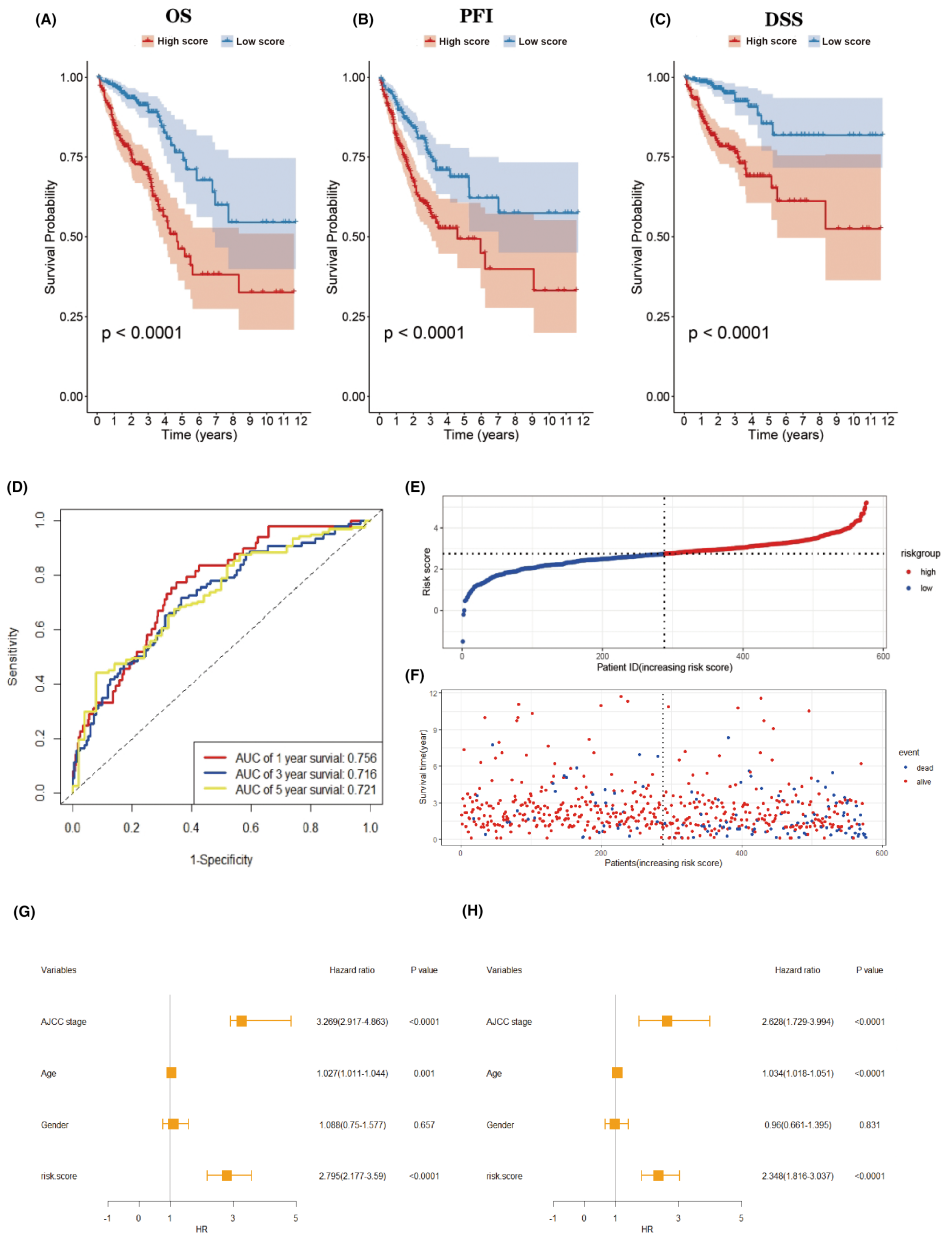
(A–C) Kaplan–Meier plot comparing the OS, PFI, DSS between the high‐score and low‐score groups. (D) ROC curves showing the 1, 3, and 5‐year AUC value in the training cohort. (E–F) Risk score and survival distribution of training group. (G–H) Univariate and multivariate Cox regression analysis of the risk score and each clinical feature in the training cohort.

Five hundred and seventy six patients in the training cohort were separated into two groups (288 high‐score patients and 288 low‐score patients) on the basis of the median value of the risk scores. Patients in the low‐score group were characterized with better OS, progression‐free interval (PFI), and disease‐specific survival (DSS) outcomes compared to patients in the high‐score group (Figure 3A–C). In addition, the 1, 3, and 5‐year AUC value were 0.756, 0.716, and 0.721, respectively, indicating a reliable accuracy evaluation of the model (Figure 3D). Every patient's survival time, status, and risk score were demonstrated using risk curves and scatter plots (Figure 3E,F). Univariate Cox regression results revealed that except for sex, all other clinical signatures and risk scores were correlated with OS (Figure 3G). Moreover, the multivariate Cox regression analysis results indicated that AJCC stage, age, and risk scores were independent risk factors (*P* < 0.05; Figure 3H).

**FIGURE 4:**
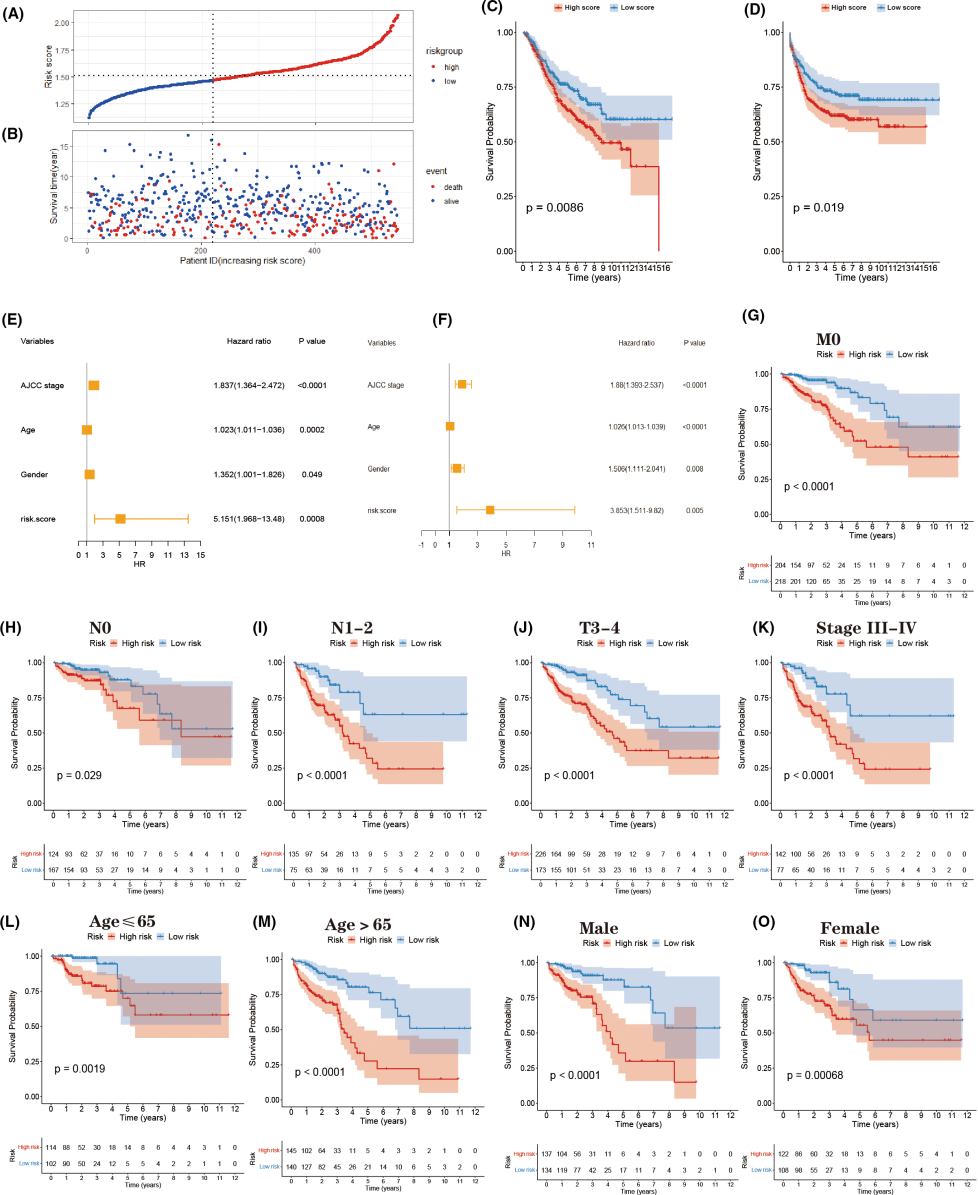
(A–B) Risk score and survival distribution in the testing dataset. (C‐D) Kaplan–Meier plot comparing the OS, RFS between the high‐score and low‐score groups. (E–F) Univariate and multivariate Cox regression analysis of the risk score and each clinical feature in the testing dataset. (G–O) Kaplan–Meier plots of different clinical features in the training cohort.

We verified the prognostic value of GRL signature in the GSE39582 dataset. The survival time, status, and risk score of each patient were demonstrated using risk curves and scatter plots (Figure 4A,B). After dividing the test cohort into two groups with 325 high‐scoring patients and 220 low‐scoring patients, survival analyses were applied. Similar to the results in the TCGA cohort, patients in the low‐score group exhibited better OS and recurrence‐free survival (RFS) compared with patients in the high‐score group (Figure 4C,D). Moreover, univariate and multivariate Cox regression analysis indicated that the risk score was also an independent risk factor in the GSE39582 dataset (Figure 4E,F).

We selected 501 patients in the training cohort with complete clinical information (TNM classification, age, sex, and clinical stage) and the distribution of the different clinical signatures and risk scores are listed in Table 1. Kaplan–Meier curves showed that the risk model exhibited excellent predictive ability in the following groups: T3–T4, M0, N0, N1–2, Stage III–IV, age ≤ 65 years, age > 65 years, women, and men (Figure 4G–O).

**TABLE 1:** Risk scores and clinicopathological features in the TCGA cohort

		Risk level expression	
	Total (*N* = 501)	High(*N* = 259)	Low (*N* = 242)
Gender			
FEMALE	230	122	108
MALE	271	137	134
Age			
≤65	216	114	102
>65	285	145	140
TNM stage			
Stage I‐II	282	117	165
Stage III‐IV	219	142	77
T			
T1‐T2	102	33	69
T3‐T4	399	226	173
M			
M0	422	204	218
M1	79	55	24
N			
N0	291	124	167
N1‐N2	210	135	75

### 3.4 Nomogram construction

**FIGURE 5:**
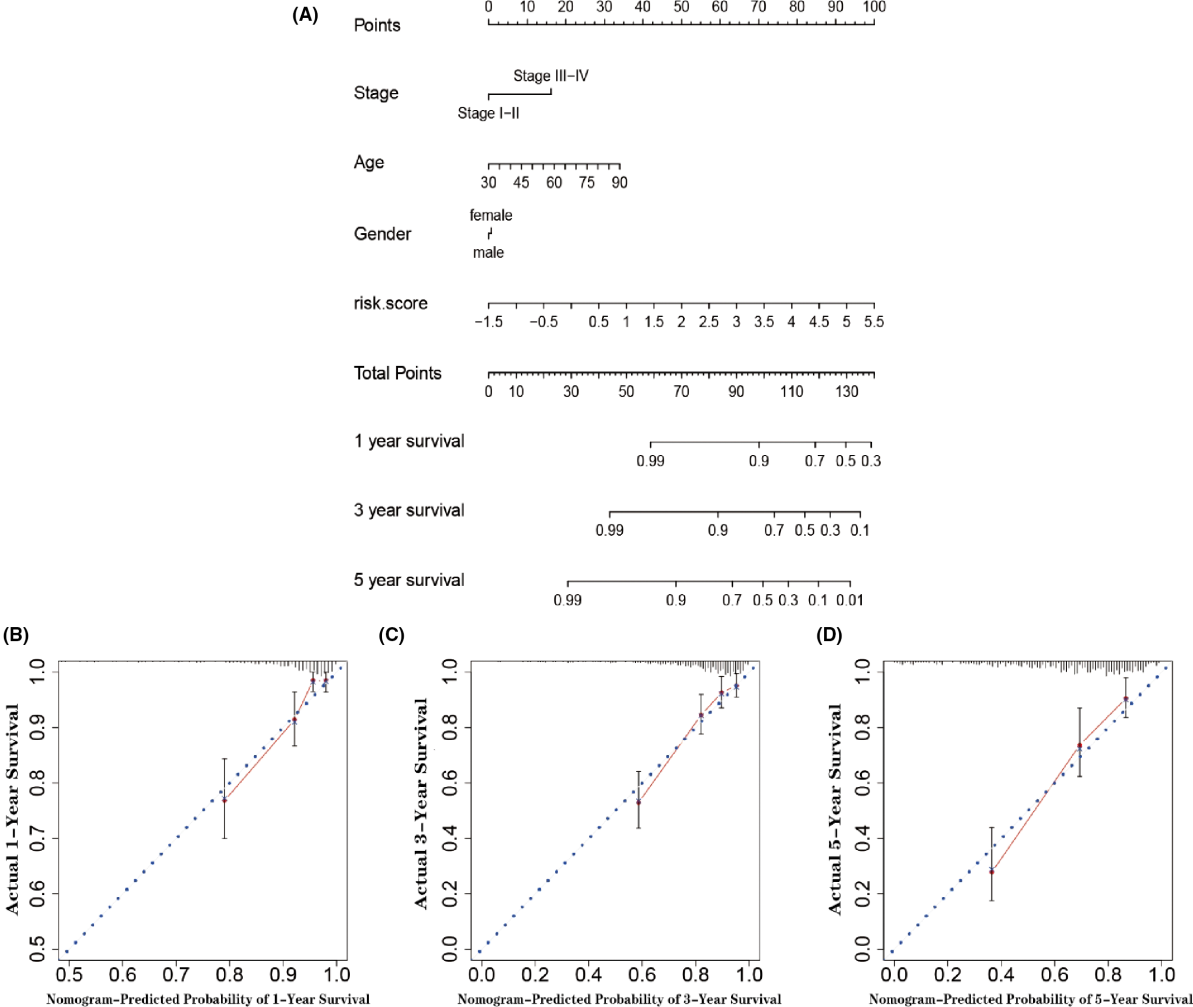
(A) Nomogram which includes age, gender, clinical stage, and risk score in the TCGA cohort. (B–D) Calibration plots were used to indicate how the nomogram effectively predicts the overall survival of CRC patients in the training group.

Risk scores and various clinicopathological features including AJCC stage, age, and gender were applied to construct a nomogram to predict the 1, 3, and 5‐year survival of patients in the training cohort (Figure 5A). A C‐index of 0.77 was calculated according to multiple Cox regression results. The calibration curves were shown in Figure 5B–D, which suggested that the constructed nomogram had excellent discrimination and accuracy.

### 3.5 Functional enrichment analysis

**FIGURE 6:**
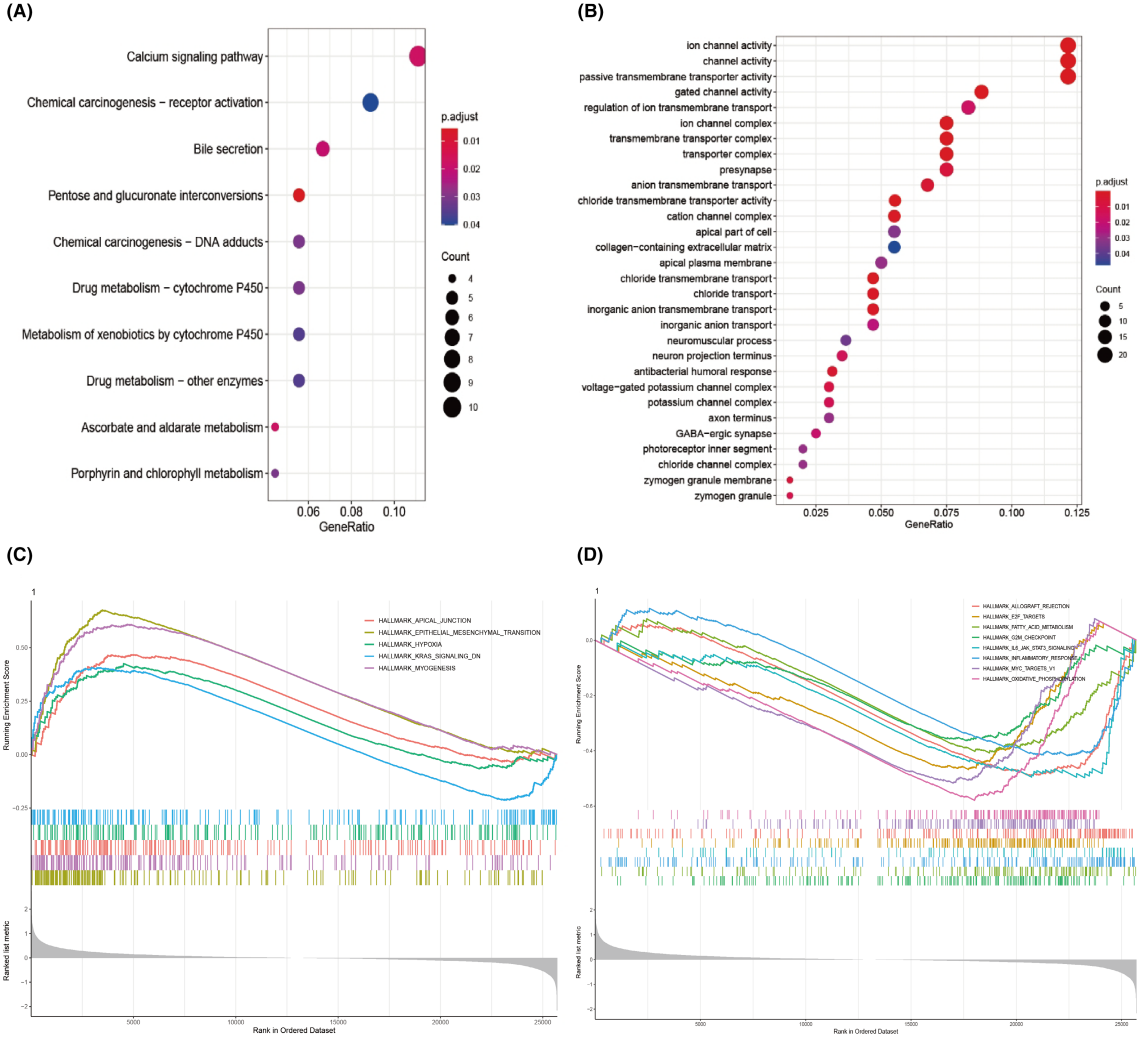
KEGG, GO, GSEA analysis. (A–B) Significantly enriched KEGG and GO pathways of DEGs between the high and low‐risk groups (p value cutoff =0.05, q value cutoff = 0.2). (C) Gene sets enriched in high‐risk group. (D) Gene sets enriched in low‐risk group.

The differentially expressed genes between the high‐ and low‐risk groups in the TCGA cohort were subjected to KEGG and GO analyses. The calcium signaling pathway was the top term found in KEGG analysis, whereas gated channel activity, passive transmembrane transporter activity, channel activity, and ion channel activity were more predominant based on the GO analysis (Figure 6A,B). Several studies have revealed that the dysregulation of ion channels, especially the calcium ion channel, is common in various human cancers and is involved in numerous important aspects of tumor‐aggressive phenotypes.^30^, ^31^ For the GSEA analysis, gene set “h.all.v7.4.entrez.gmt” was used to further investigate the possible biological behaviors of the two groups. Thirteen hallmarks were selected upon setting the significant criteria at nominal *P* value < 0.05, FDR *Q* value < 0.25, and |normalized enrichment score| > 1 (Table 2) (Figure 6C,D). Hallmarks enriched in high‐risk subgroup were mainly relevant to EMT, hypoxia, and KRAS signaling, which are crucial pathways in the progression of CRC (Figure 6C).

**TABLE 2:** GSEA results in the TCGA cohort

Name	Set size	Enrichment score	NES	*P* value	*P*.adjust	*Q* values	Rank	Leading_edge
HALLMARK_EPITHELIAL_MESENCHYMAL_TRANSITION	200	0.67549	2.439449	0.00146	0.015773	0.00963	3482	tags = 55%, list = 14%, signal = 47%
HALLMARK_MYOGENESIS	200	0.609187	2.200003	0.00146	0.015773	0.00963	4708	tags = 49%, list = 18%, signal = 40%
HALLMARK_APICAL_JUNCTION	200	0.46771	1.689077	0.00146	0.015773	0.00963	4918	tags = 38%, list = 19%, signal = 31%
HALLMARK_HYPOXIA	200	0.42569	1.537325	0.00146	0.015773	0.00963	4518	tags = 30%, list = 18%, signal = 25%
HALLMARK_KRAS_SIGNALING_DN	200	0.407549	1.471811	0.00438	0.019907	0.012154	2956	tags = 28%, list = 11%, signal = 25%
HALLMARK_G2M_CHECKPOINT	200	−0.36354	−1.40909	0.009464	0.036399	0.022223	8083	tags = 47%, list = 31%, signal = 32%
HALLMARK_FATTY_ACID_METABOLISM	158	−0.40792	−1.53063	0.008902	0.036399	0.022223	6978	tags = 54%, list = 27%, signal = 40%
HALLMARK_INFLAMMATORY_RESPONSE	200	−0.41823	−1.62107	0.003155	0.015773	0.00963	3254	tags = 30%, list = 13%, signal = 26%
HALLMARK_IL6_JAK_STAT3_SIGNALING	87	−0.49744	−1.71257	0.002604	0.015773	0.00963	2456	tags = 37%, list = 10%, signal = 33%
HALLMARK_E2F_TARGETS	200	−0.46781	−1.81326	0.003155	0.015773	0.00963	7975	tags = 57%, list = 31%, signal = 40%
HALLMARK_ALLOGRAFT_REJECTION	200	−0.48941	−1.89696	0.003155	0.015773	0.00963	4998	tags = 46%, list = 19%, signal = 38%
HALLMARK_MYC_TARGETS_V1	200	−0.51712	−2.00436	0.003155	0.015773	0.00963	8704	tags = 62%, list = 34%, signal = 42%
HALLMARK_OXIDATIVE_PHOSPHORYLATION	200	−0.5792	−2.24499	0.003155	0.015773	0.00963	7689	tags = 75%, list = 30%, signal = 53%

**TABLE 3:** Sequences of siRNA and primers used in this study

siRNA	
AFAP1‐AS1‐si1	sense 5′‐CCUAUCUGGUCAACACGUATT‐3′ antisense 5′‐UACGUGUUGACCAGAUAGGTT‐3′
AFAP1‐AS1‐si2	sense 5′‐GGGCUUCAAUUUACAAGCATT‐3′ antisense 5′‐UGCUUGUAAAUUGAAGCCCTT‐3′
Primers	
AFAP1‐AS1‐F	5′‐ATGGGATAAGAATGGCTTGCTGTGG‐3′
AFAP1‐AS1‐R	5′‐TGGTTGGTGCGGTTGGAATAGC‐3′
β‐Actin‐F	5′‐ATCATGTTTGAGACCTTCAACA‐3′
β‐Actin‐R	5′‐CATCTCTTGCTCGAAGTCCA‐3′

### 3.6 Distinct immune microenvironment features can be found between the two groups

**FIGURE 7:**
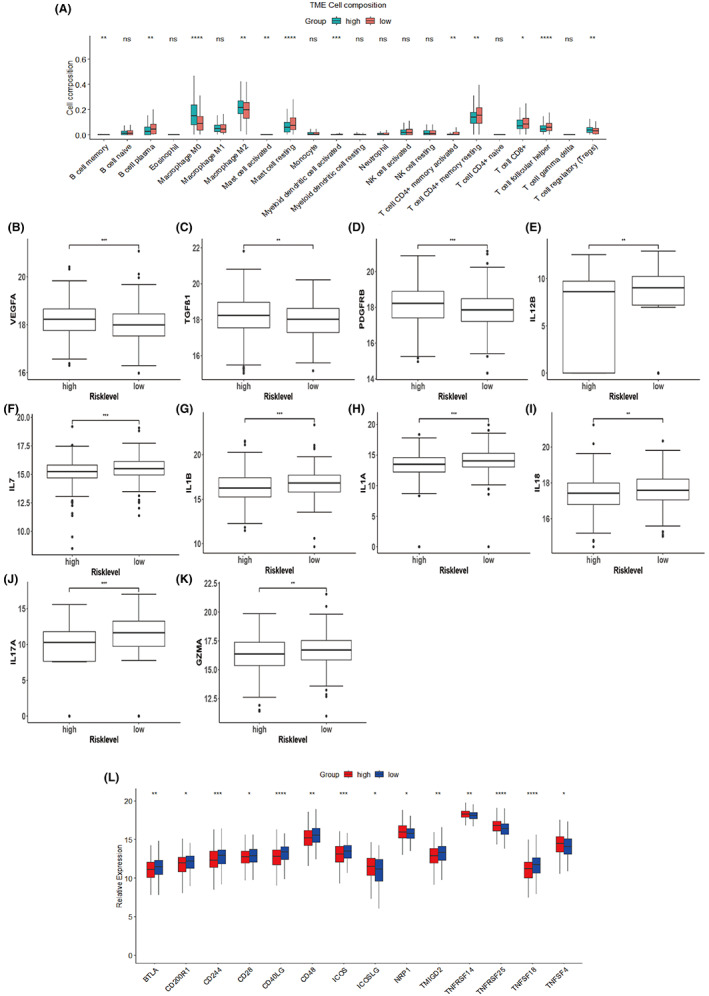
Immune infiltration analyses. (A) The proportions of 22 types of immune cells in the low‐risk (red) and high‐risk (green) group were compared using the Wilcoxon rank‐sum test (*p < 0.05, **p < 0.01, ***p < 0.001, ****p < 0.0001). (B–K) Expression of genes related to the immune response. (L) Comparison of immune checkpoint expression between the two subgroups.

The functional analysis showed a connection between the GRL signature and immune activity in CRC patients (Table 2). Moreover, proportions of immune cells that have immunosuppressive functions such as Macrophage M2 and T cell regulatory (Tregs) were significantly higher in the high‐risk group (Figure 7A). Meanwhile, in the low‐risk group, higher proportions of anti‐tumor immune cells, such as T cell CD8+, T cell follicular helper, B cell plasma, T‐cells‐CD4‐memory‐resting, and T cell CD4+ memory activated were observed (Figure 7A). Next, the expression patterns of immune‐related cytokines were analyzed. VEGFA, TGFβ1, and PDGFRB were upregulated in the high‐risk group, while pro‐inflammatory cytokines including IL12B, IL7, IL1B, IL1A, IL18, IL17A, and GZMA were higher in the low‐risk group (Figure 7B–K). Furthermore, a series of immune checkpoints were differentially expressed between the two groups (Figure 7L). Overall, these results indicate that immune suppression may contribute to the poor survival outcomes in the high‐score group.

### 3.7 Relationship between risk score and gene mutation

**FIGURE 8:**
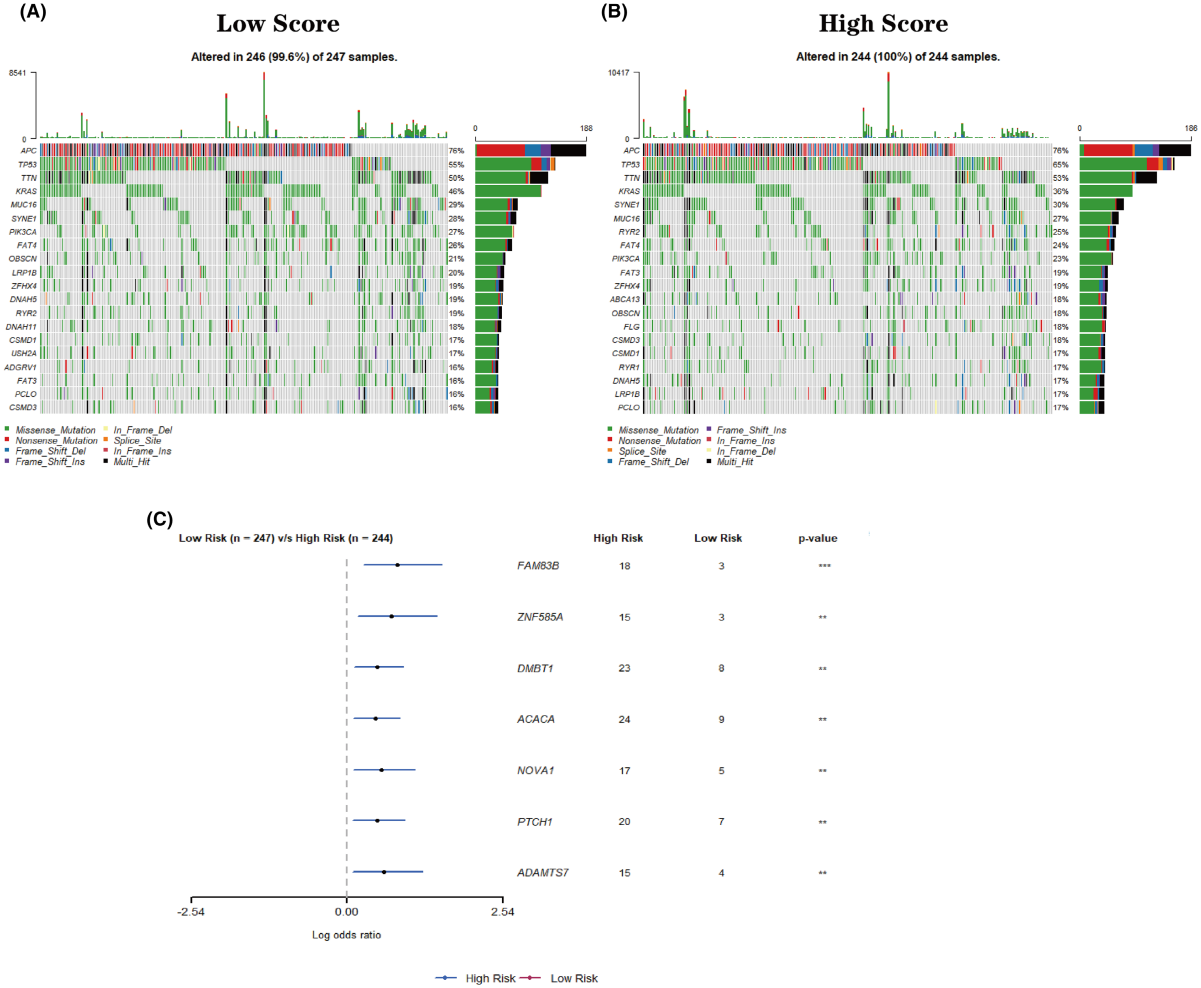
Mutation frequency of the top 20 driver genes in low‐score (A) and high‐score group (B). Forest plot of mutation genes in TCGA cohort (C).

We compared the mutation frequencies of the top 20 driver genes in each group. The waterfall plot was used to visualize the mutational landscape. In the high‐risk group, TP53 mutation frequency was higher than in the low‐risk group (65% vs. 55%; Figure 8A,B). On the contrary, only 36 percent of the patients in the high‐risk group have a KRAS mutation, which is lower than its counterpart (46%; Figure 8A,B). Subsequently, we selected genes that showed a mutation in more than 15 patients in any one group. As shown in Figure 8C, the mutation frequencies of FAM83B, ZNF585A, DMBT1, ACACA, NOVA1, PCTH1, and ADAMTS7 were significantly higher in the high‐risk group, suggesting that these mutations may contribute to tumor development.

### 3.8 Chemosensitivity difference between the two groups provides potential therapeutic targets

**FIGURE 9:**
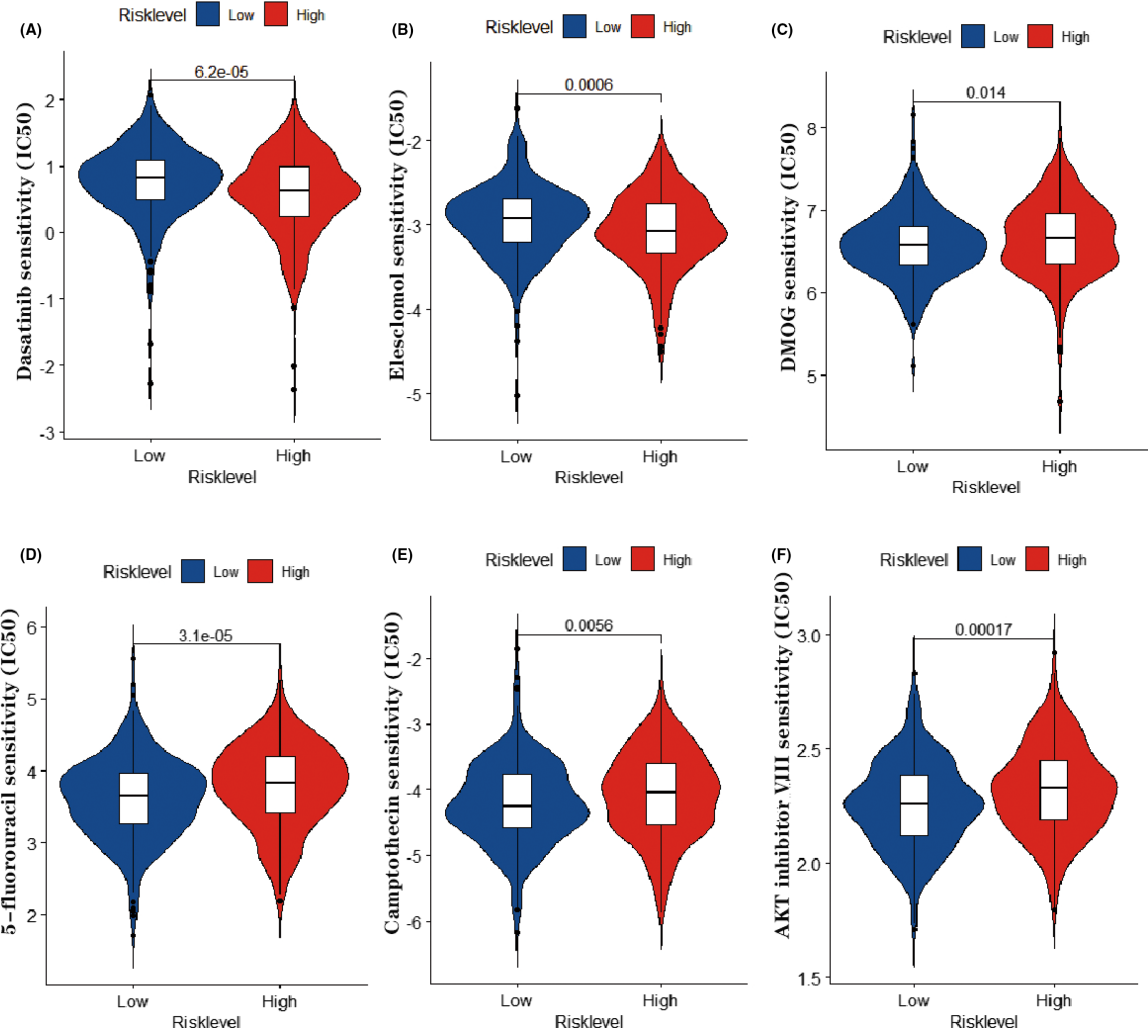
The chemosensitivity of high and low‐risk groups. IC50 of (A) dasatinib, (B) elesclomol, (C) DMOG, (D) 5‐fluorouracil, (E) camptothecin and (F) AKT inhibitor VIII between two groups was visualized using violin plot.

Chemotherapy is crucial in the treatment of CRC.^3^ However, drug resistance can occur which can further lead to the progression of cancer.^32^ We calculated the IC50 of 5‐fluorouracil, elesclomol, DMOG, dasatinib, camptothecin, and AKT inhibitor VIII of patients in training dataset. In the high‐risk group, the IC50 of dasatinib and elesclomol were significantly lower, demonstrating that patients in this group might obtain more therapeutic benefit from the two drugs (Figure 9A,B). By contrast, in the low‐risk group, the IC50 of 5‐fluorouracil, DMOG, camptothecin, and AKT inhibitor VIII were observed to be much lower (Figure 9C–F).

### 3.9 AFAP1‐AS1 knockdown inhibits the invasion, metastasis, and aerobic glycolysis of CRC cells

**FIGURE 10:**
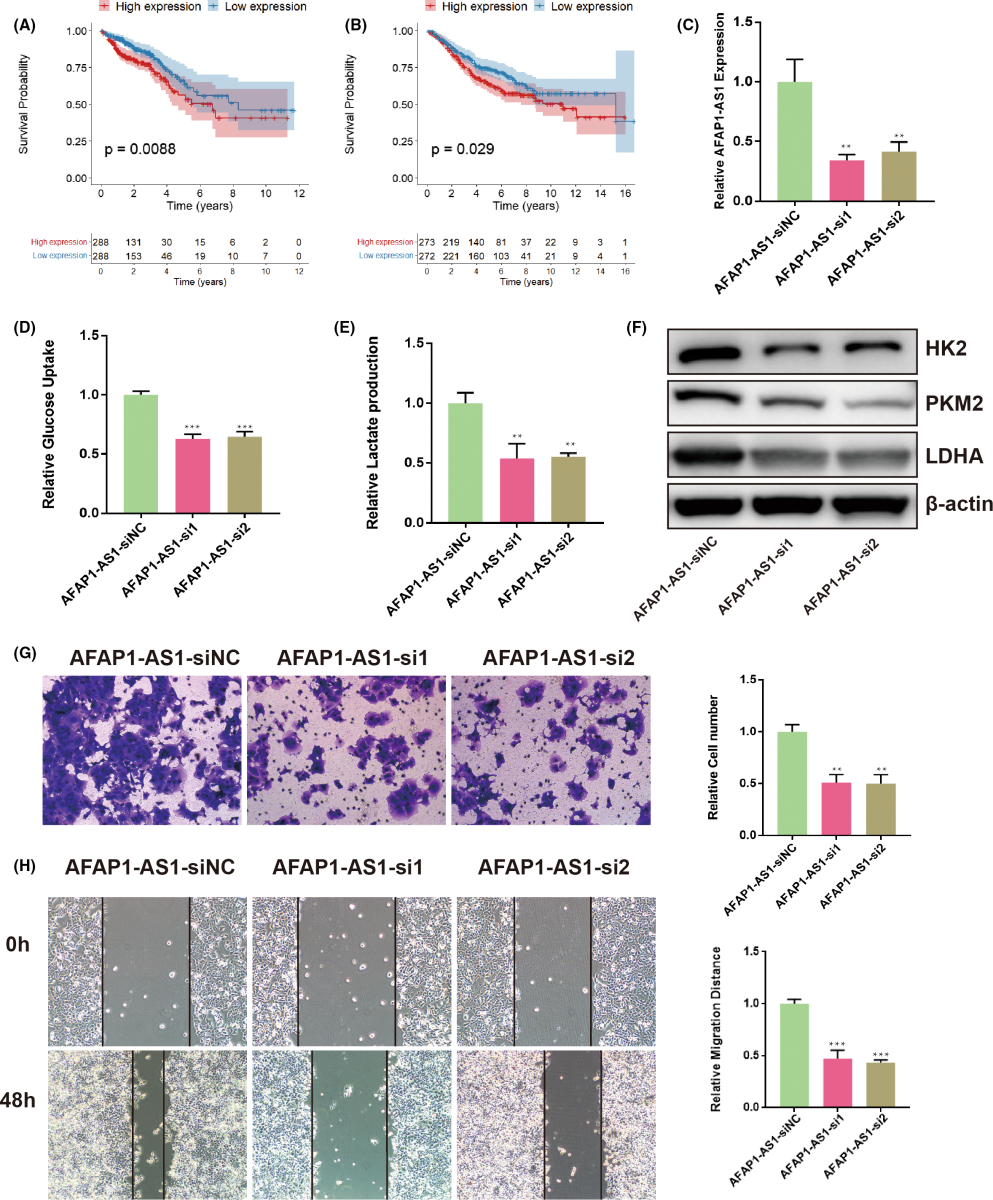
In vitro experiments. (A–B) High expression of AFAP1‐AS1 indicates worse OS in both TCGA and GSE39582 datasets. (C) Efficiency of si‐AFAP1‐AS1 was detected by RT‐qPCR. (D–E) Glucose uptake assay and lactate production assay in DLD1 cells that were transfected with siAFAP1‐AS1 or siNC. (F) Protein level of HK2, PKM2, LDHA after knocking down AFAP1‐AS1. (G) Effects of AFAP1‐AS1 on the invasion ability of DLD1 cells were measured by transwell assay. (H) Effects of AFAP1‐AS1 on the migration ability of DLD1 cells were detected by wound healing assay.

Both TCGA and GSE39582 cohorts were separated into high and low expression groups respectively on the basis of the medium value of each lncRNA. Kaplan–Meier analysis indicated that high expression of AFAP1‐AS1 in both TCGA and GSE39582 datasets was correlated with a worse OS (Figure 10A,B). AFAP1‐AS1 is found to be an oncogenic lncRNA in multiple types of cancers.^33^ For instance, AFAP1‐AS1 can promote tumor progression in CRC by enhancing zeste homolog 2 activity and may serve as a novel prognostic marker.^34^ Its metabolic functions, however, are largely unknown. Therefore, we selected AFAP1‐AS1 for further experimental analyses. The efficiency of two siRNA was verified using RT‐qPCR (Figure 10C). The glycolytic activity of DLD1 cells were significantly inhibited after knocking down AFAP1‐AS1, which was further confirmed by western blot (Figure 10D–F). In addition, AFAP1‐AS1 knockdown abolished the invasion and metastasis ability of DLD1 cells (Figure 10G,H). Collectively, these results suggested that AFAP1‐AS1 could promote the invasion, metastasis, and glycolytic ability of CRC cells, and may contribute to cancer progression by regulating aerobic glycolysis.

## 4 DISCUSSION

CRC cells undergo metabolic reprogramming to promote their survival in the TME which usually has a limited resource for nutrition and energy.^35^ The Warburg effect promotes ATP production and the synthesis of biomacromolecules at a faster rate in cancer cells to meet their urgent growth requirements.^15^, ^35^ Multiple researches have discovered that lncRNAs contribute to CRC development by activating glycolysis signaling.^36^ In a study by Wang et al., LINRIS has been shown to enhance MYC‐mediated glycolysis in CRC cells by maintaining the stability of IGF2BP2.^37^ Similarly, lncRNA GLCC1, whose expression increases following glucose deprivation, could upregulate glycolytic rates in CRC cells to impart a survival advantage.^38^ A recent study revealed that lncRNA FEZF1‐AS1 could lead to an increase in aerobic glycolysis to promote growth and metastasis in CRC.^39^ Furthermore, the expression levels of these lncRNAs are correlated with the survival outcomes of CRC patients.^37^, ^38^, ^39^

To identify and construct a GRL signature, we obtained the expression matrix and corresponding clinical data of CRC samples from the TCGA database. We selected differentially expressed lncRNAs between paracancerous and cancerous samples to limit the scope of biological marker screening. Using Pearson analysis, 315 differentially expressed GRLs were identified. Considering that lncRNA transcripts can differ between the two datasets, we only selected shared lncRNAs to ensure the replicability of the lncRNA signature. Then, a series of bioinformatic analyses were applied to deduce a risk score formula. Eventually, a 9‐lncRNA based formula with excellent predictive ability was set up.

Among the 9 lncRNAs which were identified, MIR31HG has been reported to promote glycolytic activity and oral cancer progression by co‐activating HIF‐1a.^40^ In colorectal cancer, MIR31HG has been shown to accelerate aerobic glycolysis by sponging miR‐361‐3p.^41^ Previous research found that high level of MIR31HG was correlated with shorter RFS in CRC, which is consistent with our findings.^42^ Meanwhile, HAND2‐AS1 exerts anti‐tumor functions and its expression is downregulated in multiple types of cancers.^43^ Additionally, HAND2‐AS1 has been shown to inhibit aerobic glycolysis and the proliferation of gastric cancer and osteosarcoma.^44^, ^45^ The effect of WT1‐AS in different types of can™cers is inconsistent.^46^ The expression of WT1‐AS is abnormally elevated in CRC, acute myelocytic leukemia, Wilms tumor, and breast cancer, while is downregulated in gastric cancer, liver cancer, cervical cancer, and kidney cancer.^46^ This is consistent with our findings that WT1‐AS is a risk factor in CRC. The role of LINC00996 in CRC has been systematically explored in a study, indicating that LINC00996 is downregulated in CRC and its downregulation is associated with unsatisfactory prognosis and tumor progression.^47^ Upregulated expression of AC129507.1 has been reported to the associated poor prognosis in stomach adenocarcinoma, which is contrary to what we found in CRC.^48^ FLJ16779 was applied to construct a prognostic model in gastric cancer.^49^ However, information regarding the role of LMNTD2‐AS1 and KLRK1‐AS1 in cancer development is still unknown. AFAP1‐AS1 is a rising star in lncRNA and its function has been summarized in some excellent reviews.^33^, ^50^ In our study, we found that AFAP1‐AS1 also has a metabolic impact on CRC.

To test the prognostic value of the GRL model, patients were separated into high and low‐score groups. Kaplan–Meier survival and ROC analysis demonstrated that the GRL signature could predict OS with a reliable accuracy. Cox regression analyses confirmed that this signature is an independent risk factor, and exhibits excellent predictive ability in most subgroups (T3–T4, M0, N0, N1–2, Stage III–IV, age ≤ 65 years, age > 65 years, women, and men). Lastly, a nomogram was constructed and we validated the discrimination and calibration of the model.

Functional enrichment analyses provide insights into the difference of molecular mechanisms between the two groups. The dysregulation of ion channels has been reported to be involved in most malignancies.^31^, ^51^ In addition, ionic immune suppression within TME limits the anti‐tumor effects of immune cells, leading to drug resistance in immune therapy.^52^ In this study, differentially expressed genes exhibited significant enrichment in ion channel‐related pathways, suggesting that glycolysis and ion channels may cooperate to promote cancer progression. A hypoxic TME, the main cause of glycolysis, can reshape the metabolism of cancer and impair immune cell fitness and effector functions.^53^ KRAS mutation is an emerging biomarker for conferring resistance to anti‐EGFR treatment and may serve as a promising target in advanced CRC.^54^, ^55^ Recent findings have shown that KRAS‐signaling also leads to immune suppression in CRC.^56^ Moreover, the expression of glucose transporter 1 (GLUT1) can be regulated by KRAS to promote glycolytic metabolism in cancer.^57^ EMT confers increased tumor‐initiating and metastatic potential to cancer cells and higher resistance to several therapeutic regimens.^58^ CMS4 tumors seem to exhibit a mesenchymal phenotype and show worse survival outcomes.^59^ GSEA results revealed that several pathways enriched in the high‐risk group are important for tumor progression and glycolytic metabolism. In contrast, we observed that the immune‐related pathways and oxidative phosphorylation‐related pathways enriched in the low‐risk group indicated better survival rates and lower glycolytic levels. The immune analysis also shows that the two groups in the TCGA dataset exhibit different immune phenotypes. Immune suppression is an important factor leading to tumor development. Mutations of oncogenes and tumor suppressor genes lead to the abnormal metabolism of CRC.^13^ Our results showed that a higher mutation frequency of TP53 can be found in the high‐risk group. TP53 is an important tumor suppressor gene that helps maintain the metabolic homeostasis of cells, and its mutation leads to an enhanced level of glycolysis in tumor cells.^60^

Tumor heterogeneity makes precision medicine very important in overcoming drug resistance.^61^ IC50 calculation based on expression matrices can predict the sensitivity of every patient to various drugs. It has been found that a 5‐fluorouracil‐based chemotherapy regime is an indispensable part of CRC treatment.^3^ Our results suggest that patients in the high‐score group are predicted to be more resistant to 5‐fluorouracil‐based therapy. Hence, our model may provide a predictive value in guiding therapeutic strategies for CRC patients.

Several limits exist in this research. The detailed molecular mechanism of the 9 GRLs is poorly understood and should be investigated further. Additionally, a third cohort (own cohort) is not available in this research. More specifically, future studies should be focused on identifying the exact molecular mechanisms underlying lncRNA functions that lead to poor survival in patients with CRC. In summary, we constructed a GRL signature on the basis of 9 GRLs. This signature is correlated with patients' survival and clinicopathological features. Furthermore, the relevant signaling pathways and immune infiltration landscape as identified by our model may pave the way for new therapeutic strategies for CRC. Finally, we demonstrated that AFAP1‐AS1 could regulate aerobic glycolysis and be a promising target in CRC.

## AUTHOR CONTRIBUTION

Xinyang Zhong, Xuefeng He and Yaxian Wang had an equal contribution to this manuscript. Dawei Li, Ping Wei and Hong Zhang designed the whole study. Xinyang Zhong and Xuefeng participated in the experiments, bioinformatics and statistical analyses. Zijuan Hu, Yaxian Wang and Senlin Zhao wrote the original draft. Yaxian Wang and Huixia Huang designed the figures and tables in this study. Dawei Li and Ping Wei revised the manuscript. All authors approved this manuscript.

## FUNDING INFORMATION

This research was supported by grants from the National Science Foundation of China (81772583, 81972293, 81972185) and Shanghai Rising‐Star Program (19QA1402200).

## CONFLICT OF INTEREST

The authors declare that they have no competing interests.

## ETHICS APPROVAL AND CONSENT TO PARTICIPATE

Not applicable.

## Data Availability

In this study, all the data can be found here: https://www.cancergenome.nih.gov/, https://www.ncbi.nlm.nih.gov/geo/query/acc.cgi?acc=GSE39582, http://www.gsea‐msigdb.org/gsea/msigdb/, http://timer.comp‐genomics.org/,UCSC Xena (https://xena.ucsc.edu/).
